# The *MC4R* SNPs, their haplotypes and gene-environment interactions on the risk of obesity

**DOI:** 10.1186/s10020-020-00202-1

**Published:** 2020-08-08

**Authors:** Bi-Liu Wei, Rui-Xing Yin, Chun-Xiao Liu, Guo-Xiong Deng, Yao-Zong Guan, Peng-Fei Zheng

**Affiliations:** 1 Department of Cardiology, Institute of Cardiovascular Diseases, the First Affiliated Hospital, Guangxi Medical University, 6 Shuangyong Road, Nanning, Guangxi 530021 People’s Republic of China; 2 Guangxi Key Laboratory Base of Precision Medicine in Cardio-cerebrovascular Disease Control and Prevention, Nanning, Guangxi 530021 People’s Republic of China; 3 Guangxi Clinical Research Center for Cardio-cerebrovascular Diseases, Nanning, Guangxi 530021 People’s Republic of China

**Keywords:** Melanocortin 4 receptor gene (*MC4R*), Single nucleotide polymorphism, Obesity, Environmental factor

## Abstract

**Background:**

Little is known about the correlation between the melanocortin 4 receptor gene (*MC4R*) single nucleotide polymorphisms (SNPs) and the risk of obesity. This research sought to test the *MC4R* rs17782313, rs476828 and rs12970134 SNPs, their haplotypes and gene-environment interactions on the risk of obesity in the Maonan ethnic group, an isolated minority in China.

**Methods:**

A case-control study comprised of 1836 participants (obesity group, 858; and control group, 978) was conducted. Genotypes of the three SNPs were determined by the next-generation sequencing (NGS) technology.

**Results:**

The genotypic frequencies of the three SNPs were different between the obesity and control groups (*P* <  0.05 for all). The minor allelic frequency of the *MC4R* rs17782313C, rs476828C and rs12970134A was higher in obesity than in control groups (13.8% vs. 8.3%, *P* <  0.001, 17.1% vs. 10.9%, *P* <  0.001; and 15.5% vs. 11.5%, *P* <  0.001; respectively). Additionally, the dominant model of rs17782313 and rs476828 SNPs revealed an increased morbidity function on the risk of obesity (*P* <  0.05). A correlation between SNP-environment and the risk of obesity was also observed. The rs17782313C-rs476828C-rs12970134A haplotype was associated with high risk of obesity (OR = 1.796, 95% CI = 1.447–2.229), whereas the rs17782313T-rs476828T-rs12970134G and rs17782313T-rs476828T-rs12970134A haplotypes were associated with low risk of obesity (OR = 0.699, 95% CI = 0.586–0.834 and OR = 0.620, 95% CI = 0.416–0.925; respectively). The interactions between haplotype and waist circumference on the risk of obesity were also noted.

**Conclusions:**

We discovered that the *MC4R* rs17782313, rs476828 and rs12970134 SNPs and their haplotypes were associated with the risk of obesity in the Chinese Maonan population.

## Introduction

Obesity represents a serious global health problem (Doak et al. 2012). More than 0.4 billion people all over the world are obese based on the criteria designated by the World Health Organization. In addition, several studies have suggested that obesity is associated with increased risks of type 2 diabetes, cardiovascular disease and hypertension (Dixon 2010).

Obesity is a complex disease that is modified by an interaction between genetic and environmental factors (Xi et al. 2011). Recent research on the genetic elements that place individuals at risk of obesity has uncovered several contributing factors. A genome-wide association study (GWAS) from 2008 reported that the melanocortin 4 receptor gene (*MC4R*) was associated with obesity (Loos et al. 2008). Another study found that the rs17782313 polymorphism, located near the *MC4R*, is linked to corpulency in European adults and children (Loos et al. 2008). Two polymorphisms (rs12970134 and rs476828) near the *MC4R* have also been associated with increased risk of obesity in Europeans and Europeans, respectively (Thorleifsson et al. 2009; Grant et al. 2009). Additionally, several research papers have also demonstrated *MC4R* variability across different ethnic groups (Grant et al. 2009; Hotta et al. 2009; Tabara et al. 2009; Cauchi et al. 2009; Renstrom et al. 2009; Zobel et al. 2009; Meyre et al. 2009; Willer et al. 2009; Cheung et al. 2010; Shi et al. 2010; Huang et al. 2011; Rouskas et al. 2012; Beckers et al. 2011; Thomsen et al. 2012; Tao et al. 2012; Liem et al. 2010; Wu et al. 2010; Vogel et al. 2011; Ng et al. 2010), although some studies fail to demonstrate any significant correlation (Grant et al. 2009; Hotta et al. 2009; Tabara et al. 2009; Liem et al. 2010; Ng et al. 2010). The differences may be owed to the modest impact of polymorphism, the limited statistical ability of small sample sizes, and the discrepancy in the genetic and environmental factors of the study subjects.

China has 56 ethnic groups. According to the statistics of the sixth national census in 2010, Han Chinese represents the largest population. The Maonan nationality is a minority group of Southeastern China and consists of approximately 107,166 people. The closest anthropological cousins of the Maonan are the Buyi ethnic group (Ogata et al. 2007). In Guangxi, the genetic correlation between the Maonan population and other minorities is much higher than that between the Maonan and Han (Deng et al. 2007; Yao et al. 2009). A previous study has demonstrated that the three *MC4R* (rs17782313, rs12970134 and rs476828) SNPs have an impact on obesity, but their relationship to the risk of obesity has yet to be clearly outlined. Therefore, the objective of this research was to determine the relationship between the three *MC4R* SNPs, their respective interactions with the environment and the obese phenotype in the Maonan people. Our research utilizes the multi-dimensional dimensionality reduction (MDR) method to analyze the correlation between the *MC4R* SNPs based on haplotype clustering and gene × environment (G × E) interactions on obesity in the Maonan population.

## Materials and methods

### SNP selection

Three SNPs of *MC4R* were chosen by the following steps: (1) They were tagging SNPs identified by Haploview (Broad Institute or MIT and Harvard, Cambridge, MA, USA, version 4.2). (2) *MC4R* rs17782313, rs476828 and rs12970134 SNPs were then selected by SHEsis Main (http://analysis.bio-x.cn/myAnalysis.php). (3) The minor allele frequency (MAF) of the SNPs was more than 1%. (4) The SNPs may be potentially associated with obesity in the previous researches. (5) Related data of the SNPs could be gained from NCBI dbSNP Build 132 (http://www.ncbi.nlm.nih.gov/SNP/).

### Subjects

This is a case-control study (Batarfi et al. 2019) and a total of 1836 Maonan individuals which were previously included in stratified stochastic specimens were randomly selected (Guo et al. 2016). All individuals were living in the Huanjiang Maonan Autonomous County, which is located in Northwestern Guangxi Zhuang Autonomous Region of China. The individuals’ age ranged from 18 to 90 (mean 55.38 ± 15.33) years in controls. Ages < 40, 40–49, 50–59, 60–69, 70–79 and ≥ 80 years were 155 (15.8%), 206 (21.1%), 209 (21.4%), 196 (20.0%), 158 (16.2%) and 54 (5.5%) people; respectively. The participants’ age ranged from 22 to 90 (mean 56.29 ± 14.25) years in patients with obesity. Ages < 40, 40–49, 50–59, 60–69, 70–79 and ≥ 80 years were 132 (15.4%), 184 (21.4%), 186 (21.7%), 171 (19.9%), 139 (16.2%) and 46 (5.4%) persons; respectively. The selection criteria for Maonan individuals have been described in detail in two previous epidemiological studies (Wang et al. 2016; Bin et al. 2017). In addition, all Maonan subjects were also confirmed by Y chromosome and mitochondrial diversity studies. All individuals were healthy, they had no evidence of related diseases such as diabetes, atherosclerosis, and coronary heart disease. The participants were also not on any medications known to influence obesity. The sample size was calculated using QUANTO software (http://hydra.usc.edu/gxe) (Wang and Li 2004). All people in the participating population gave written informed consent to participate in epidemiological investigations and genetic analysis. All research protocols on this topic have been approved by the Ethics Committee of the First Affiliated Hospital of Guangxi Medical University (No. Lunshen-2014 KY-Guoji-001, Mar. 7, 2014).

### Epidemiologic investigation

The epidemiologic investigation used international standardization methods and an established study protocol (Zhang et al. 2017). The study used standardized questionnaires to collect information regarding demographics, socioeconomic status, and lifestyle factors. Smoking or drinking status was designated into either one of two groups (yes or no) (Li et al. 2018). The research also incorporated several parameters to measure blood pressure, waist circumference (WC) and other clinical procedures. Body mass index (BMI, kg/m^2^) was calculated based on preexisting formulas.

### Biochemical measurements

All participants fasted for at least 12 h prior to venous blood sampling. The values of total cholesterol (TC), triglyceride (TG), high-density lipoprotein (HDL-C), and low-density lipoprotein cholesterol (LDL-C) in specimens were measured using commercially available enzyme assay kits, CHOL2 for TC, TRIGL for TG, HDLC4 for HDL-C, and LDLC3 for LDL-C (Roche Diagnostics GmbH., Sandhofer Strasse 116, 68,305 Mannheim, Germany). The detected ranges of TC, TG, HDL-C and LDL-C were 0.1–20.7, 0.1–10.0, 0.08–3.88 and 0.10–14.2 mmol/L or 3.86–800, 8.85–885, 3.09–150 and 3.87–549 mg/dL, respectively. Furthermore, the detection limits of the CHOL2, TRIGL, HDLC4 and LDLC3 Kits were 0.1 mmol/L, 0.1, 0.08  and 0.1 mmol/L, or 3.86, 8.85, 3.09 and 3.87 mg/dL, respectively, which are the lowest measurable analyte level (different from zero). The intra-batch precision of the assay kits was 21. An autoanalyzer (Type cobas c 701/702; Roche Diagnostics GmbH., Sandhofer Strasse 116, 68,305 Mannheim, Germany) was used to perform all determinations in the Clinical Science Experiment Center of the First Affiliated Hospital, Guangxi Medical University (Aung et al. 2014).

### Diagnostic criterion

The normal values of TC, TG, HDL-C and LDL-C in our Clinical Science Experiment Center were 3.10–5.17, 0.56–1.70, 1.16–1.42 and 2.70–3.10 mmol/L, respectively. One or more of the following conditions were used to define dyslipidemia: LDL-C ≥ 3.6 mmol/l; TG ≥ 1.7 mmol/l; HDL-C < 1.29 mmol/l in women or < 1.03 mmol/l in men; TC ≥ 5.7 mmol/l, basing on the NECP-ATP III criteria (Zhang et al. 2012; Grundy et al. 2006). Hyperlipidemia depended on TC > 5.17 mmol/L, and/or TG > 1.70 mmol/L (Wu et al. 2016). Hypertension was determined when the participants have a systolic blood pressure of 140 mmHg or greater, and/or a diastolic blood pressure of 90 mmHg or higher (Chalmers et al. 1999). Participants were separated into two groups, according to age > 60 or ≤ 60. WC was defined as ≥90 cm for men or ≥ 80 cm for women subgroups (Grundy et al. 2006; Saely et al. 2006). A BMI of < 23 and ≥ 25 kg/m^2^ was determined as control and obesity (Wen et al. 2009); respectively.

### DNA amplification and genotyping

Genomic DNA was separated from leucocytes of venous blood using the phenol-chloroform method (Miao et al. 2018). All separated DNA samples were stored at 4 °C until further analysis. Genotyping of the three SNPs was accomplished by next-generation sequencing technology in the NGS department, Sangon Biotech (Shanghai) Co; Ltd. **(1) Sense and antisense primers:** the sense and antisense primers used were 5′-TCCACATGCTATTGGTTTAAGACAAGTC-3′ and 5′-TCCCAGATGCTAAAATGATACTCCTCAATA-3′ for the rs17782313; 5′-ACTTCTTAGTCTGGCTGTCAACAAAC-3′ 5′-CCCTTGGAGTAGCAATTGGAAG-3′ for the rs476828; and 5′-GAGACTGGCAAAGCAGAGTTTT-3′ and 5′-CCTTTGAATACAGGTACTAACAAGCAC-3′ for the rs12970134; respectively. **(2) Multiplex PCR and sequencing:** A panel which contains 3 target SNPs (rs17782313, rs476828 and rs12970134) was designed. Library preparation was performed by two-step PCR. First round PCR reaction was set up: DNA (10 ng/μl) 2 μl; amplicon PCR forward primer mix (10 μM) 1 μl; amplicon PCR reverse primer mix (10 μM) 1 μl; 2 × PCR Ready Mix 15 μl (total 25 μl, Kapa HiFi Ready Mix). The plate was sealed and PCR performed in a thermal instrument (BIO-RAD, T100TM) using the following program: 1 cycle of denaturing at 98 °C for 5 min, first 8 cycles of denaturing at 98 °C for 30 s, annealing at 50 °C for 30 s, elongation at 72 °C for 30 s, then 25 cycles of denaturing at 98 °C for 30 s, annealing at 66 °C for 30 s, elongation at 72 °C for 30 s and a final extension at 72 °C for 5 min. Finally hold at 4 °C. The PCR products were checked using electrophoresis in 1% (w/v) agarose gels in TBE buffer (Tris, boric acid, EDTA) stained with ethidium bromide (EB) and visualized under UV light. Then we used AMPure XP beads to purify the amplicon product. After that, the second round PCR was performed. PCR reaction was set up: DNA (10 ng/μl) 2 μl; universal P7 primer with barcode (10 μM) 1 μl; universal P5 primer (10 μM) 1 μl; 2 × PCR Ready Mix 15 μl (total 30 μl, Kapa HiFi Ready Mix). The plate was sealed and PCR performed in a thermal instrument (BIO-RAD, T100TM) using the following program: 1 cycle of denaturing at 95 °C for 3 min, then 5 cycles of denaturing at 94 °C for 30 s, annealing at 55 °C for 20 s, elongation at 72 °C for 30 s, elongation at 72 °C for 30 s and a final extension at 72 °C for 5 min. Then we used AMPure XP beads to purify the amplicon product. The libraries were then quantified and pooled. Paired-end sequencing of the library was performed on the HiSeq XTen sequencers (Illumina, San Diego, CA). **(3) Data QC and SNP calling:** raw reads were filtered according to two steps: 1) removing adaptor sequence if reads contains by cutadapt (v 1.2.1); 2) removing low quality bases from reads 3′ to 5′ (Q < 20) by PRINSEQ-lite(v 0.20.3); and the remaining clean data were mapped to the reference genome by BWA (version 0.7.13-r1126) with default parameters. A perl script was written to calculate each genotype of target site. Annovar (2018-04-16) was used to detect genetic variants.

### Statistical analyses

The data was statistically analyzed using the statistical software SPSS 22.0 (SPSS Inc., Chicago, IL, USA). Normally distributed quantitative data were expressed in terms of mean ± SD, whereas TG for non-normally distributed data was represented in terms of medians and interquartile ranges. Qualitative data was presented in terms of percentage and was analyzed using the Chi-square test between the obesity and control groups. The student’s unpaired *t*-test was used to test the general characteristics which are normally distributed between two groups. The difference in TG levels between the two groups was detected by the Wilcoxon-Mann-Whitney test. Analysis of genotype, allele and haplotype distribution between two groups was tested by the chi-squared test. The Hardy-Weinberg equilibrium (HWE), Pair-wise linkage disequilibrium (LD) and frequencies of haplotype were calculated by the SHEsis Main software (http://analysis.bio-x.cn/myAnalysis.php) (Shi and He 2005). Using the SHEsis software, *D*′ and *r*^2^ were used to detect pair-wise LD patterns between selected variants. Unconditional logic regression was used to test both the correlation of genotypes (common homozygote genotype =1, heterozygote genotype = 2, rare homozygote genotype = 3), alleles (the minor allele non-carrier = 1, the minor allele carrier = 2) and haplotypes (the haplotype non-carrier = 1, the haplotype carrier = 2) with the risk hazard of obesity, but also the SNP- or haplotype- environment interactions on the risk hazard of obesity after gender, age, WC, smoking, alcohol consumption, hypertension and hyperlipidemia were adjusted (Li et al. 2018; Miao et al. 2018). Related risks were evaluated by odds ratio (OR) and 95% confidence interval (95% CI), and *P* <  0.05 was regarded as statistical significance. The best interactive combination between the SNPs, haplotypes and environmental factors (WC, age, smoking, drinking and sex) was screened by generalized multifactor dimensionality reduction (GMDR) (Lou 2015; Lou et al. 2007), which is a free, open-source interaction analysis tool. It is enriched in options for detecting gene-gene and gene-environment interaction in different design, such as case-control design. For case-control design, by default, GMDR can detect interactions for unrelated individuals. The GMDR software is entirely available at website (http://www.soph.uab.edu/ssg/software or http://ibi.zju.edu.cn/software) (Xu et al. 2016). The cross-validation consistency score (also known as 10-fold cross-validation) is a method of measuring the degree of consistency of the selected interactions, and the result is expressed in N1/N2 (N ranges from 1 to 10) (Miao et al. 2018; Lin et al. 2013). Among all the possibilities considered, the selected interactions were determined as the best model. Test balance accuracy is a measure of the degree of interaction that accurately estimates case-control status, with a score between 0.50 (indicating that the model predicts worse than chance) and 1.00 (indicating perfect prediction) (Miao et al. 2018; Lin et al. 2013). Finally, a sign test or permutation test of the prediction accuracy (providing empirical *P*-values) can be used to estimate the signification of the recognition model (Miao et al. 2018; Lin et al. 2013).

## Results

### Demographical characteristics of study population

Demographical parameters of the 1836 study subjects are summarized in Table 1. The mean values of BMI, WC, systolic blood pressure (SBP), diastolic blood pressure (DBP), TC, TG and LDL-C levels and the percentages of subjects who smoked cigarettes were higher while HDL-C value was lower in obese patients compared to the control subjects (*P* <  0.05–0.001). However, no discrepancies were noted in terms of age structure, glucose levels, sex ratio, and drinking between the two groups (*P* > 0.05 for all).

**Table 1:** Comparison of general characteristics between control and obesity groups

Parameter	Control	Obesity	*t* (x^2^)	*P*
Number	978	858		
Age (years)^a^	55.38 ± 15.33	56.29 ± 14.25	−1.323	0.186
Body mass index (kgm^2^)	20.01 ± 1.48	27.62 ± 3.07	−66.13	< 0.001
Waist circumference (cm)	71.51 ± 6.487	87.52 ± 8.14	−46.264	< 0.001
Systolic blood pressure (mmHg)	131.49 ± 23.28	137.83 ± 22.79	−5.88	< 0.001
Diastolic blood pressure (mmHg)	80.64 ± 12.36	85.57 ± 13.67	−8.063	< 0.001
Glucose (mmol/L)	6.17 ± 1.37	6.24 ± 1.32	−1.283	0.200
TC (mmol/L)	4.89 ± 0.98	5.09 ± 0.93	−4.607	< 0.001
TG (mmol/L)^b^	1.17 (0.93)	1.63 (1.22)	−14.095	< 0.001
HDL-C (mmol/L)	1.30 ± 0.29	1.25 ± 0.26	3.792	< 0.001
LDL-C (mmol/L)	3.10 ± 0.45	3.18 ± 0.50	−3.695	< 0.001
Male/female^c^	450/528	395/463	0.000	0.991
Smoking status [n (%)]				
Non-smoker	772 (78.9)	637 (74.2)		
Smoker	206 (21.1)	221 (25.8)	5.643	0.018
Alcohol consumption [n (%)]				
Non-drinker	795 (81.3)	686 (80.0)		
Drinker	183 (18.7)	172 (20.0)	0.522	0.470

*TC* Total cholesterol, *TG* Triglyceride, *LDL-C* Low-density lipoprotein cholesterol, *HDL-C* High-density lipoprotein cholesterol

^a^Mean ± SD was detected by *t*-test

^b^Because of non-normal distribution, triglyceride value was presented as median (interquartile range), the discrepancy between the two groups was detected by the Wilcoxon-Mann-Whitney test

^c^Difference of classified Data was determined as Chi-square test

### Genotype and allele frequencies and their respective associations with obesity

As shown in Table 2, the genotype and minor allele frequencies of the rs17782313, rs476828 and rs12970134 SNPs were different between the obesity and control groups (*P* <  0.05). All mutations exhibited HWE (*P* > 0.05 for all). At the same time, the morbidity functions of the rs17782313 and rs476828 SNPs were increased in the dominant model (*P* <  0.05), whereas there was no statistical significance in the dominant mode of rs12970134 SNP (*P* > 0.05).

**Table 2:** The correlation between the *MC4R* polymorphisms and the risk of obesity [n (%)]

SNP	Genotype	Control (*n* = 978)	Obesity (*n* = 858)	χ^2^	*P*	Adjusted OR (95% CI)	*P
rs17782313 T > C	TT	823 (84.2)	633 (73.8)	29.974	< 0.001	1	–
	CT+ CC	155 (15.8)	225 (26.2)			1.428 (1.066–2.061)	0.019
	MAF	163 (8.3)	237 (13.8)	28.258	< 0.001		
	*P*_HWE_	0.613	0.210				
rs476828 T > C	TT	780 (79.8)	582 (67.8)	33.921	< 0.001	1	–
	CT+ CC	198 (20.2)	276 (32.2)			1.585 (1.176–2.136)	0.003
	MAF	213 (10.9)	293 (17.1)	29.431	< 0.001		
	*P*_HWE_	0.262	0.053				
rs12970134 G > A	GG	765 (78.2)	606 (70.6)	13.927	< 0.001	1	–
	AG+ AA	213 (21.8)	252 (29.4)			1.253 (0.924–1.698)	0.147
	MAF	224 (11.5)	266 (15.5)	12.961	< 0.001		
	*P*_HWE_	0.565	0.085				

*MC4R* The melanocortin 4 receptor gene, *MAF* Minor allele frequency, *HWE* Hardy-Weinberg equilibrium. *P* was detected as Chi-square test probability. *P was determined as Logistic test probability

### Haplotypes and the risk of obesity

**Fig. 1:**
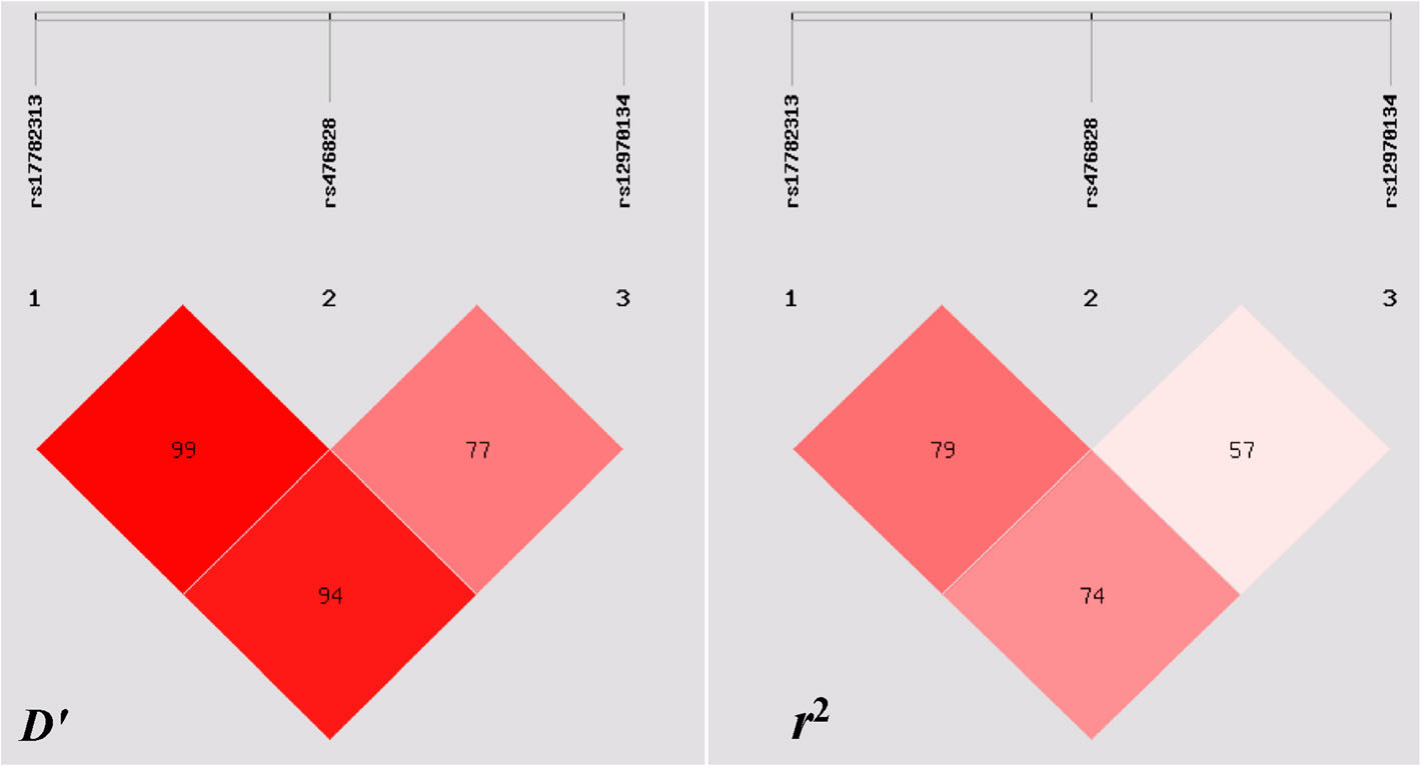
Linkage disequilibrium (LD) analysis of the *MC4R* SNPs in both populations. The LD degree was represented by pair-wise *D’* and *r*^2^

Multiple-locus LD analyses indicated that the tested sites in the study population were not statistically independent. Table 3 and Fig. 1 show strong LD between control and obesity groups (*D*′ = 0.72–0.99). As shown in Table 4, the commonest haplotypes were T-T-G, C-C-A and T-T-A (> 30% of the samples). There were significant differences in the frequency of T-T-G, C-C-A and T-T-A haplotypes between the obesity and control groups. In the meantime, conservations of the T-T-G, C-C-A and T-T-A haplotypes were observed, whereas the C-C-A haplotype contributed to an increased morbidity function (*P* <  0.05).

**Table 3:** Degree of linkage disequilibrium between the *MC4R* SNPs and the combined population of obesity and control groups

*r*^2^	rs17782313	rs476828	rs12970134
rs17782313	1		
rs476828	0.765	1	
rs12970134	0.711	0.524	1

**Table 4:** Prevalence of haplotype frequencies in the obesity and control groups [*n* (frequency)]

Haplotype	Obesity	Control	χ^2^	*P*	OR (95% CI)
C-C-A	227.77 (0.133)	153.67 (0.079)	28.845	< 0.001	1.796 [1.447–2.229]
C-C-G	9.23 (0.005)	9.31 (0.005)	–	–	–
T-C-A	0.00 (0.000)	1.01 (0.001)	–	–	–
T-C-G	56.00 (0.033)	48.99 (0.025)	1.896	0.168458	1.313 [0.890–1.938]
T-T-A	38.23 (0.022)	69.32 (0.035)	5.570	0.018294	0.620 [0.416–0.925]
T-T-G	1384.77 (0.807)	1673.67 (0.856)	15.946	< 0.001	0.699 [0.586–0.834]

The haplotype is combined with *MC4R* rs17782313-rs476828-rs12970134. Rare Hap (frequency < 3%) in both groups has been ignored in analysis

### SNP and haplotype-environment interaction on the risk of obesity

**Fig. 2:**
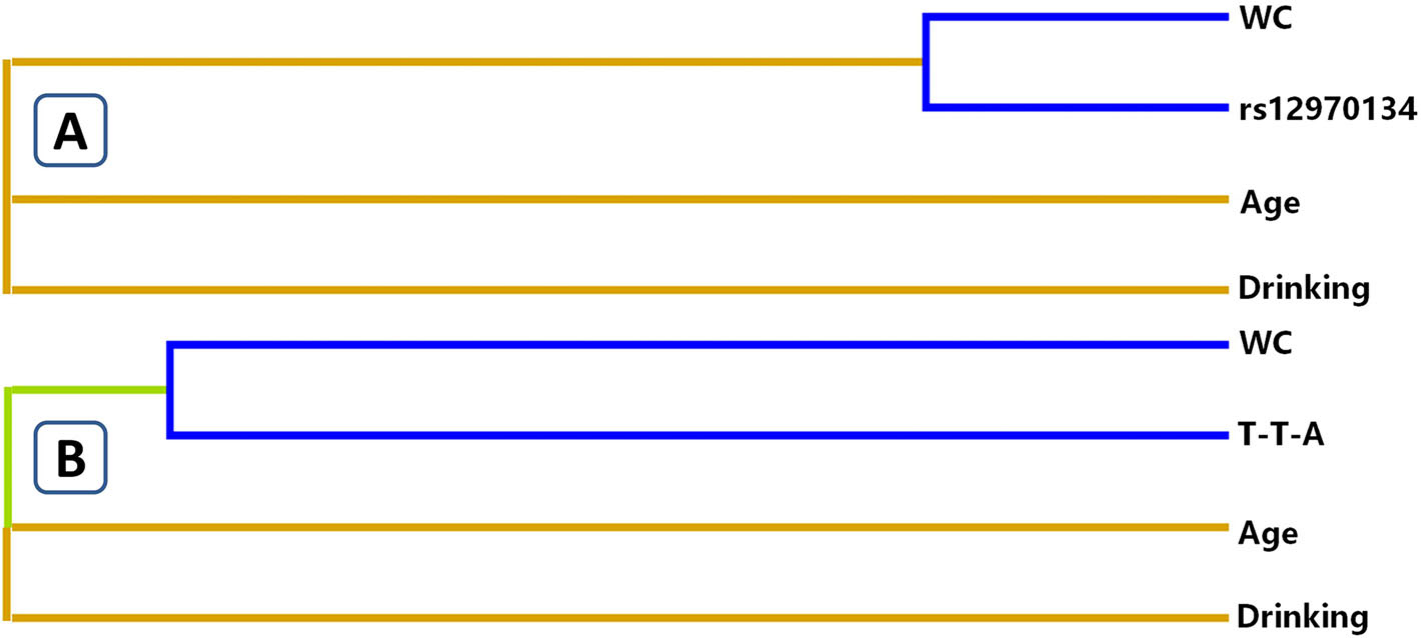
Different types of interaction dendrogram for SNP-environment (**a**) and haplotype-environment (**b**) interactions on the risk of obesity. Blue color, strong redundancy interaction; green color, weak redundancy interaction; orange color, synergy. The elements with strong interaction are close to each other at the leaves of tree, and the elements with weak interaction are far away from each other

The influence of gene-environment exposures including the interactions between SNPs, age, gender, BMI, WC, tobacco and/or alcohol consumption on obesity risk was analyzed by the GMDR model, after adjustment for covariates. Table 5 summarizes the results obtained from the GMDR analysis for two- to three-locus models for gene-environment interaction. A significant three-locus model (*P* <  0.001) involving rs12970134 SNP, drinking and WC was found, indicating a potential interaction between SNPs and these environmental factors. In the meantime, this model had a cross-validation consistency of 10 of 10, with a testing accuracy of 82.56% (Miao et al. 2018; Lin et al. 2013). Moreover, the three-locus model also tested haplotype-environment interactions (WC, drinking and T-T-A, *P* <  0.001). An entropy-based interaction dendrogram built by MDR is shown in Fig. 2, which revealed the strongest redundancy effect in the SNP-environment interaction (rs12970134 and WC) and in the haplotype-environment interaction (WC and T-T-A). In order to acquire the OR and 95%CI for the combined effects, we performed an interaction study using logistic regression analyses (Table 6). When the SNP-environment interaction was analyzed, we revealed that the participants with rs12970134 GA/AA genotypes and WC (male ≥90 cm or female ≥80 cm) had higher risk of obesity compared to the participants with rs12970134 GG and WC (male < 90 cm or female < 80 cm; adjusted OR = 95.069, 95%CI = 47.260–191.351, *P* <  0.001). In addition, when the haplotype-environment interactions were studied, we detected that the carriers of T-T-A haplotype and WC (male ≥90 cm or female ≥80 cm) had higher obesity risk than the non-carriers and WC (male ≥90 cm or female ≥80 cm; adjusted OR = 51.533, 95% CI = 12.131–218.912, *P* <  0.001).

**Table 5:** GMDR analysis of SNPs, haplotype and environment showed different interactions

Locus no.	Best combination	Training Bal. Acc	Testing Bal. Acc	Cross-validation consistency	*P*	^*^P
SNP-environment interaction						
2	WC + Age	0.8213	0.8213	10/10	< 0.001	< 0.001
3	WC+ drinking+ rs12970134	0.8256	0.8256	10/10	< 0.001	< 0.001
Haplotype-environment interaction						
2	WC+ Age	0.8213	0.8213	10/10	< 0.001	< 0.001
3	WC+ drinking+ T-T-A	0.8232	0.8232	10/10	< 0.001	< 0.001

*P*, adjusting for sex, age, smoking, drinking, hypertension, hyperlipidemia, waist circumference (WC), and impaired fasting glucose. *P, indicates 1000 permutation tests. The haplotype is combined with *MC4R* rs17782313-rs476828-rs12970134

**Table 6:** Using logistic regression to analyze different types of interactions

Variable 1	Variable 2	OR (95% CI)	*P*
SNP-environment interaction			
rs12970134	WC (male ≥90 cm, female ≥80 cm)		
GG	No	1	–
GG	Yes	42.577 (28.714–63.132)	< 0.001
GA + AA	No	1.07 (0.758–1.51)	0.701
GA + AA	Yes	95.096 (47.260–191.351)	< 0.001
Haplotype-environment interaction			
T-T-A	WC (male ≥90 cm, female ≥80 cm)		
Non-carriers	No	1	–
Non-carriers	Yes	51.371 (40.001–65.972)	< 0.001
Carriers	No	0.503 (0.246–1.027)	0.059
Carriers	Yes	51.533 (12.131–218.912)	< 0.001

*P*, adjusting for sex, age, smoking, drinking, hypertension, hyperlipidemia, and impaired fasting glucose. T-T-A is combined with *MC4R* rs17782313-rs476828-rs12970134

## Discussion

Obesity is a known contributor towards cardiovascular illness and premature mortality (Peeters et al. 2003; Jimenez et al. 2018). The occurrence of obesity is the product of interaction between a variety of environmental factors, such as diet, unhealthy lifestyle, lack of exercise as well as genetic factors (Xi et al. 2011; Unamuno et al. 2018; Teixeira et al. 2016). Biologically active mediators which are released by adipose tissue have a significant impact on weight, insulin resistance as well as changes in blood pressure and lipid levels, all of which result in endothelial dysfunction and atherosclerosis.

Current research has identified an association between the *MC4R* mutations and obesity. The genotypic and allelic frequencies of three *MC4R* SNPs were significantly different between the obesity and control participants. These outcomes strongly suggest that the prevalence of obesity may stem from genetic elements. Upon closer observation of the relationship between the *MC4R* SNPs and their haplotypes and the risk of obesity, we noted that the rs17782313C-rs476828C-rs12970134A haplotype increased the risk of obesity. Conversely, the rs17782313T-rs476828T-rs12970134G and rs17782313T-rs476828T-rs12970134A haplotypes were associated with decreased risk of obesity. At the same time, we also found that the participants with the rs12970134 GA/AA genotypes and WC (male ≥90 cm or female ≥80 cm; SNP-environment interaction) had higher risk of obesity than the individuals with rs12970134 GG and WC (male < 90 cm or female < 80 cm). The carriers of T-T-A haplotype and WC (male ≥90 cm or female ≥80 cm; haplotype-environment interaction) had higher risk of obesity than the haplotype non-carriers and WC (male ≥90 cm or female ≥80 cm). These observations underscore the strong role of genetic influences in the development of obesity (Unamuno et al. 2018; Teixeira et al. 2016; Ruixing et al. 2008).

The Maonan diet consists of large proportions of pork, animal viscus and beef, all of which are rich in saturated fatty acid (Miao et al. 2018). High-fat diets are significant contributors of obesity, dyslipidemia (especially raised plasma TC and TG levels (Lottenberg et al. 2012), atherosclerosis, and hypertension (Teixeira et al. 2016; Ruixing et al. 2008) which may explain the differences in the prevalence of hypertension, plasma TC and TG levels between the two groups at present. The percentages of subjects who consumed alcohol and smoked cigarettes were high amongst the Maonan adult population, the percentages of cigarette smoking were significantly different between the obesity and control groups, whereas there was no significant difference in the percentages of alcohol consumption between the two groups. The effects of alcohol consumption and cigarette smoking on obesity have previously been studied (Gruchow et al. 1985; Fulkerson and French 2003; Audrain-McGovern and Benowitz 2011; Seeley and Sandoval 2011; Rigotti and Clair 2018). Most smokers are underweight, and quitting smoking often leads to being overweight or obesity (Fulkerson and French 2003; Audrain-McGovern and Benowitz 2011; Seeley and Sandoval 2011; Rigotti and Clair 2018). However, several different observational studies on alcohol consumption and smoking have yielded contradictory results, warranting further investigations involving different cohorts, ethnicities and age groups across different populations (Sayon-Orea et al. 2011; Bendsen et al. 2013). Several GWASes have uncovered genetic variants that are associated to different aspects of general wellbeing. However, it is sometimes overlooked that the genetic variations found in GWAS may represent the effects of modifiable hazardous elements as well as direct genetic influences (Gage et al. 2016). Our research sought to dissect this possibility by using examples of patients who partake in high fat diets or those who had high alcohol and cigarette usage.

Our research has several limitations. Firstly, the importance of several other genetic and environmental elements cannot be discounted, for example, energy intake, physical activity and dietary patterns. Secondly, the sample size of this research is relatively small and should be expanded. Finally, obesity is undoubtedly a complex and multifactorial illness (Chooi et al. 2019). Although our studies have tested the correlation of three *MC4R* SNPs and their haplotypes to the risk of obesity, several other gene-environment interactions still need to be measured.

## Conclusions

In summary, our study investigated the potential interactions between the *MC4R* SNPs, environment and obesity in the Maonan population. Moreover, the correlation analysis based on haplotype clustering and G × E interactions may be more informative regarding the risk of obesity in contrast to single-locus tests. GMDR analysis demonstrated several different interactions that exist between gene and environment that may be able to impact patient morbidity.

## Abbreviations

BMI: Body mass index
CI: Confidence interval
DBP: Diastolic blood pressure
GMDR: Generalized multifactor dimensionality reduction
GWAS: Genome-wide association study
HDL-C: High-density lipoprotein cholesterol
HWE: Hardy-Weinberg equilibrium
LD: Linkage disequilibrium
LDL-C: Low-density lipoprotein cholesterol
MAF: Minor allele frequency
MC4R: Melanocortin 4 receptor
NGS: Next-generation sequencing
OR: Odds ratio
SBP: Systolic blood pressure
SNP: Single nucleotide polymorphism
TC: Total cholesterol
TG: Triglyceride
WC: Waist circumference
